# Assessing the internal validity of a household survey-based food security measure adapted for use in Iran

**DOI:** 10.1186/1475-2891-8-28

**Published:** 2009-06-26

**Authors:** Morteza Rafiei, Mark Nord, Atefeh Sadeghizadeh, Mohammad H Entezari

**Affiliations:** 1 Medical Education Research Center of Isfahan University of Medical Sciences, Isfahan, Iran; 2 Economic Research Service, United States Department of Agriculture, USA; 3 Health Faculty, Isfahan University of Medical Sciences, Isfahan, Iran

## Abstract

**Background:**

The prevalence of food insecurity is an indicator of material well-being in an area of basic need. The U.S. Food Security Module has been adapted for use in a wide variety of cultural and linguistic settings around the world. We assessed the internal validity of the adapted U.S. Household Food Security Survey Module to measure adult and child food insecurity in Isfahan, Iran, using statistical methods based on the Rasch measurement model.

**Methods:**

The U.S. Household Food Security Survey Module was translated into Farsi and after adaptation, administered to a representative sample. Data were provided by 2,004 randomly selected households from all sectors of the population of Isfahan, Iran, during 2005.

**Results:**

53.1 percent reported that their food had run out at some time during the previous 12 months and they did not have money to buy more, while 26.7 percent reported that an adult had cut the size of a meal or skipped a meal because there was not enough money for food, and 7.2 percent reported that an adult did not eat for a whole day because there was not enough money for food. The severity of the items in the adult scale, estimated under Rasch-model assumptions, covered a range of 6.65 logistic units, and those in the child scale 11.68 logistic units. Most Item-infit statistics were near unity, and none exceeded 1.20.

**Conclusion:**

The range of severity of items provides measurement coverage across a wide range of severity of food insecurity for both adults and children. Both scales demonstrated acceptable levels of internal validity, although several items should be improved. The similarity of the response patterns in the Isfahan and the U.S. suggests that food insecurity is experienced, managed, and described similarly in the two countries.

## Background

Food security – consistent access to adequate food for active, healthy living [1] – is an important foundation for good nutrition and health. An estimated 20% of the Iranian population suffers from energy and protein insufficiency [2]. This problem can affect the quality of life of households [3,4]. In Iran, several indirect indicators including income and various methods have been used to estimate the extent of food insecurity [5,6]. Zerafati et al modified the Radimer/Cornell questionnaire to measure food insecurity in low-income urban households in Tehran, the capital of Iran. They found high levels of food insecurity in the sample and some support for the validity and reliability of the instrument, but concluded that further modifications were necessary to reliably measure food insecurity at the household level [6].

During the 1990s, household survey-based methods for assessing food security were developed in the United States [7-9]. These methods have since used for annual monitoring of household food security in the United States since 1995 [10]. Recent researches have demonstrated the validity of HFSSM tool as an inexpensive, easy to use and analyzing method for evaluating the actual level of food insecurity [11]. They have also been adapted for use in a wide variety of cultural and linguistic contexts around the world, and have generally demonstrated both internal and external validity [12-16]. In 2006, researchers at the Isfanhan University of Medical Sciences adapted the U.S. module for Iranian population, translated it into Farsi, and administered it to a representative sample of households in Isfahan, Iran.

In this article, we assessed the internal validity of measures of household-level adult and child food insecurity based on the Isfahan Food Security Survey data. We determined the performance of each item and of the adult and child measures of food insecurity using statistical methods based on the Rasch measurement model [17-21]. The Rasch Model provides a theoretical statistical framework for inferring the associations of items with a latent trait based on the observed associations among the items. [22]. We examined the extent to which the Isfahan food security scales appear to measure the same phenomenon as is measured in the U.S. using similar methods. This comparison explores the extent to which the phenomenon of food insecurity is similar in these two distinct cultural and linguistic groups.

## Methods

### The U.S. Household Food Security Survey Module (US-HFSSM)and food security scale

The US-HFSSM is a measure of the severity of household food access problems. It is based on self-reported behaviors, experiences, and conditions collected by interviewing one member of each household using a standardized survey instrument – the US-HFSSM [23]. The food security status of each household is assessed by their responses to 18 questions (10 in households with no children) about food-related behaviors, experiences, and conditions that are known to characterize households having difficulty meeting their food needs. The questions cover a wide range of severity of food access problems ranging from worrying about running out of food to children not eating for a whole day. The questions have been developed from anthropological and case-study research among low-income U.S. families regarding their experiences of food deprivation and how they described and coped with them [24-26]. The questions reflect familiar conditions, experiences, and behaviors, and use natural language derived from the qualitative research to describe them. Each question specifies a lack of money or other resources to obtain food as the reason for the condition or behavior, so the scale is not affected by hunger due to voluntary dieting or fasting. In the standard module, all questions are referenced to the previous 12 months, although shorter time references (e.g., 30 days) are also practical.

Responses to the questions in the food security survey module are combined into a scale using non-linear statistical methods based on the Rasch measurement model. The scale provides a continuous, graduated measure of the severity of food deprivation across the range of severity represented by the items. Based on their food security scale scores, households are also classified into food security status categories for monitoring and statistical analysis of the food security status of the population.

### Adaptation the US-HFSSM for the Isfahan Food Security Survey

To adapt original module according to Iranian culture, a focus group including a number of nutritionists and sociologists was formed.

The focus group was to consider Iranian eating habits and culture and to find close – to -Iranian – culture equivalent terms in order to translate the original questionnaire to Farsi. The survey module was translated into Farsi and a back-translated into English and then it was examined for consistency with the original. The questions were re-ordered so that the child-referenced questions were all grouped together following the adult-referenced questions (a change that will also be implemented in the U.S. module in future national surveys).

The original English version of the questions is included in table 1.

**Table 1:** The Original English Version of the Questions

**Adult Questions:**	
AD1.	I'm going to read you several statements that people have made about their food situation. For these statements, please tell me whether the statement was often true, sometimes true, or never true for (you/your household) in the last 12 months – that is, since last (name of current month). The first statement is "(I/We) worried whether (my/our) food would run out before (I/we) got money to buy more." Was that often true, sometimes true, or never true for (you/your household) in the last 12 months?
AD2.	"The food that (I/we) bought just didn't last, and (I/we) didn't have money to get more." Was that often, sometimes, or never true for (you/your household) in the last 12 months?
AD3.	"(I/we) couldn't afford to eat balanced meals." Was that often, sometimes, or never true for (you/your household) in the last 12 months?
AD4.	In the last 12 months, since last (name of current month), did (you/you or other adults in your household) ever cut the size of your meals or skip meals because there wasn't enough money for food? (Yes/No)
AD4b.	[IF YES ABOVE, ASK] How often did this happen – almost every month, some months but not every month, or in only 1 or 2 months?
AD5.	In the last 12 months, did you ever eat less than you felt you should because there wasn't enough money to buy food? (Yes/No)
AD6.	In the last 12 months, were you every hungry but didn't eat because there wasn't enough money for food? (Yes/No)
AD7.	In the last 12 months, did you lose weight because there wasn't enough money for food? (Yes/No)
AD8.	In the last 12 months, did (you/you or other adults in your household) ever not eat for a whole day because there wasn't enough money for food? (Yes/No)
AD8b.	[IF YES ABOVE, ASK] How often did this happen – almost every month, some months but not every month, or in only 1 or 2 months?

**Child Questions:**	

CH1.	"(I/we) relied on only a few kinds of low-cost food to feed (my/our) child/the children) because (I was/we were) running out of money to buy food." Was that often, sometimes, or never true for (you/your household) in the last 12 months?
CH2.	"(I/We) couldn't feed (my/our) child/the children) a balanced meal, because (I/we) couldn't afford that." Was that often, sometimes, or never true for (you/your household) in the last 12 months?
CH3.	"(My/Our child was/The children were) not eating enough because (I/we) just couldn't afford enough food." Was that often, sometimes, or never true for (you/your household) in the last 12 months?
CH4.	In the last 12 months, since (current month) of last year, did you ever cut the size of (your child's/any of the children's) meals because there wasn't enough money for food? (Yes/No)
CH5.	In the last 12 months, did (CHILD'S NAME/any of the children) ever skip meals because there wasn't enough money for food? (Yes/No)
CH5b.	[IF YES ABOVE ASK] How often did this happen – almost every month, some months but not every month, or in only 1 or 2 months?
CH6.	In the last 12 months, (was your child/were the children) ever hungry but you just couldn't afford more food? (Yes/No)
CH7.	In the last 12 months, did (your child/any of the children) ever not eat for a whole day because there wasn't enough money for food? (Yes/No)

### Data

Data were provided by 2,004 randomly selected households from all sectors of the Isfahan population during 2005, using the adapted and translated questionnaire.

One household provided no responses to any of the food security questions and was omitted from the analysis. For the remaining 2,003 households, item-specific missing data were rare. Only 34 households (1.6 percent) had any missing responses to the adult questions, and 28 of those missed only a single question. Of the 990 households with valid responses to the child-referenced questions, only 11 missed any questions and 8 of those missed only a single question. The most frequently missed questions were the three "how often?" follow up questions (AD4b, AD8b, and CH5b).

### Analytic methods

We constructed and assessed separate scales for adult food security and child food security rather than a single scale combining adult and child items. The combined scale has proved problematic for some research and monitoring purposes in the United States because the relationship between children's and adults' food insecurity in the same household depends to a great extent on the ages of children [27]. Preliminary analysis confirmed that adult and child food insecurity also represented distinct dimensions in the Isfahan Food Security Survey data.

Responses were coded into 10 adult items and 8 child items following standard methods used in the U.S.. For the often/sometimes/never responses, "often" or "sometimes" were coded as affirmative (value = 1), and "never" was coded as negative (value = 0). For yes/no responses, "yes" was coded as 1 and "no" as 0. For "how often?" responses, "almost every month" and "some months" were coded as 1 and "only 1 or 2 months" was coded as 0. The "how often?" follow up items were coded 0 if the base item (i.e., response to the preceding question) was 0, and missing if the base item was missing.

Based on an initial examination of response patterns, we omitted the three "how often" follow up questions, questions, AD4b, AD8b, and CH5b, from further analysis and from the proposed adult and child scales. These questions added little information to differentiate levels of severity of food insecurity because they were practically redundant with their base questions. For example, of those reporting that adults had ever cut the size of meals or skipped meals in the previous 12 months, only 4 percent reported that this had occurred in only 1 or 2 months. The corresponding proportion for adults not eating for a whole day was 13 percent and for children skipping meals, 7 percent.

We then fitted the remaining 8 adult items and, in a separate analysis, the 7 child items to the Rasch model using WINSTEPS software [28]. We examined the item-infit statistics to assess whether the items all measured the same underlying condition with approximately equal discrimination. Item-infit is an information-weighted misfit statistic that assesses the logistic association of each item with the underlying condition measured by the set of items. In effect, it compares the strength of that association for each item with the average for all items. The expected value is 1, and higher values indicate weaker associations (i.e., misfit). We also examined item-outfit (outlier sensitive) statistics, which are similar to item-infit statistics but are based on squared errors and are, therefore, sensitive to highly improbable responses. A high outfit statistic indicates one or more erratic responses which may arise from misunderstanding of the item by the respondent or miscoding by the interviewer or may indicate an item that has a different meaning or relates to food insecurity differently for a small proportion of respondents.

Then, for each scale, we assessed conditional independence of items by extracting principal components from the correlation matrix of the standardized residuals. The residual is calculated for each item-respondent pair as the difference between the response (0 or 1) and the probability of an affirmative response given the calibration (i.e., the severity) of the item and the measured severity of food insecurity of the household. Each residual is standardized by dividing by the square root of its variance. (The variance is calculated as *pq*, where *p *is the probability of an affirmative response and *q *is the probability of a negative response.) Principle components factor analysis was then conducted based on the correlations of the standardized residuals across households.

Finally, we compared the item calibrations (severity parameter estimates) with those for corresponding items in the U.S. Current Population Survey Food Security Supplement data. The severity parameter of an item is the level of severity of food insecurity at which households typically switch from denying the item to affirming it. In a subsample of households with food insecurity equal to that of the severity of a specific item, half would affirm the item and half would deny it. This comparison provides information on the extent to which food insecurity is experienced, managed, and described similarly in the two countries. If the relative severity of items is similar in the two surveys, then household severity levels and prevalence rates may be meaningfully compared between the two surveyed populations.

## Results

### Adult scale

The range of severity of items was evident in the proportions of households affirming each item (table 2). Just over half of the households (53.1 percent) reported that their food had run out at some time during the previous 12 months and they did not have money to buy more, while 26.7 percent reported that an adult had cut the size of a meal or skipped a meal because there was not enough money for food. The frequency of affirmative response to items decreases as the severity of the conditions referenced by them increases.

**Table 2:** Response characteristics, item calibrations, and item-fit statistics of items in the Isfahan Adult Food Security Scale

Item*	Affirmative responses (%)**	Item calibration***	SE****	Item infit	Item outfit
AD1 Worried food would run out	48.7	3.45	0.078	0.76	1.85
AD2 Food ran out; did not have money for more	53.1	2.86	0.085	0.95	1.47
AD3 Could not afford to eat nutritious meal	50.9	3.17	0.081	0.97	4.20
AD4 Cut size of meal or skipped meal	26.7	5.90	0.077	1.03	1.80
AD5 Ate less than thought he/she should	21.3	6.58	0.084	0.90	1.62
AD6 Hungry but did not eat	13.2	7.93	0.101	0.76	0.81
AD7 Lost weight	10.3	8.60	0.113	1.09	2.03
AD8 Did not eat for whole day	7.2	9.51	0.134	1.05	1.14
					
Mean		6.00			
Standard deviation		2.44			
Discrimination parameter		1.00			
Number of cases*****	2,003	1,241	1,241	1,241	1,241

*The full wording of each question specified the time reference (previous 12 months) and specified that the condition occurred because of a lack of money for food.

**Percent affirmative responses(%) of all households

***Item calibration indicates the severity of the item. The calibrations for the adult scale were estimated on a logistic scale (that is, with discrimination equal to 1). The zero point of the adult scale (which is arbitrary) was set such that the mean of item scores was 6.0, a value which ensures that all item scores and all household scores are greater than zero.

****Standard error of estimation of item calibration

*****The scaling analysis sample omits households that affirmed no items or that affirmed all items, since those responses do not provide information about the severity of the items relative to one another.

Item calibrations estimated under Rasch-model assumptions ranged from 2.86 to 9.51. The calibrations for the adult scale were estimated on a logit scale (that is, with a discrimination parameter of 1). For a group of households with the same level of severity of food insecurity, the difference between the calibrations of two items corresponds to the predicted log of the odds ratio of affirmative responses to the two items. The zero point of a Rasch model-based scale is arbitrary. For the adult scale, the zero point was set such that the mean of item scores was 6.0, a value which ensured that all item scores and all household scores would be greater than zero. Standard errrors of estimation for most items were less than 0.1 logit, with the largest 0.134 logits.

Item-infit statistics indicate that all items measure the same underlying condition. Most were near unity, and none exceeded 1.30, which would indicate an item too weakly associated with food insecurity to be included in the measure [29].

Responses by a very small proportion of households were responsible for the high outfit statistics. Cross tabulation of item responses by raw score indicated that the highest outfit (AD3) resulted from responses of just 8 households out of the total scaling sample of 1,241. The next three highest outfits (AD1, AD4, and AD7) each resulted from responses of about 4 households (but different households for each item).

**Figure 1:**
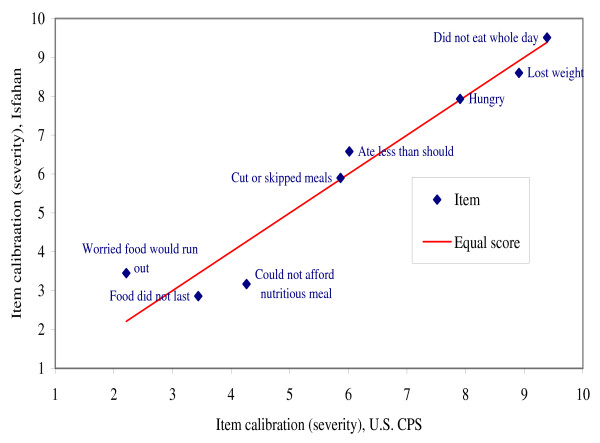
**Comparison of calibrations (severity parameter estimates) of adult items in the Isfahan Food Security Survey versus the U.S. Current Population Survey Food Security supplement**. Note: For this comparison, the calibrations of the items estimated from the U.S. CPS-FSS data were adjusted by a linear transformation to equate the mean and standard deviation of the item calibrations to those estimated from the Isfahan data.

Figure 1 plots the item severity scores for the Isfahan Food Security Survey data against those from the U.S. Current Population Survey Food Security Supplement (U.S. CPS-FSS). With the exception item AD1 (Worried food would run out), the items are in the same severity order in the two surveys. The general phenomenon of food insecurity appears to be experienced and described similarly in these two populations.

The assessment of conditional independence, or dimensionality, indicated an unexpectedly high correlation among residuals of items AD1 and AD2 ("worried food would run out" and "food did not last"; analysis not shown). With that exception, there was no indication of problematic multi-dimensionality.

The overall fit of the Isfahan data to the Rasch model is similar to that of the same items in the U.S. CPS-FSS. The standard deviation of the 8 item calibrations (2.44) was essentially the same as the standard deviation of the corresponding item calibrations in the U.S. scale [1].

The measured range of the Isfahan 8-item adult scale is from 2.38 (raw score = 1) to 9.57 (raw score = 7), a total range of 7.19 units (table 3) (The tabled value of 10.51 for raw score 8 is not included in the measured range. Technically, the score for households that said "yes" to all items cannot be determined without additional assumptions about the distribution of food insecurity in the population. They are more food insecure than those with raw score 7, but the size of the interval cannot be determined based only on item calibarations. The tabled score is an approximation based on a hypothetical raw score of 7.5.) Taking into consideration the standard error of measurement (which is a function of the number of items in the scale and their discrimination), the measured range is sufficient to differentiate three ranges of severity.

**Table 3:** Measured values of severity of adult food insecurity by raw score on Isfahan adult food security scale, and prevalence of adult food insecurity among households in the Isfahan food security pilot survey

Raw score	Household score*	Measurement error	Percent of households	Cumulative percent of households
0	Unknown**	NA	34.95	34.95
1	2.38	1.20	9.84	44.78
2	3.62	1.08	9.39	54.17
3	4.84	1.12	19.92	74.09
4	6.07	1.09	8.49	82.58
5	7.20	1.05	5.79	88.37
6	8.31	1.06	4.49	92.86
7	9.57	1.23	4.04	96.90
8	10.51**	1.57	3.10	100.00

* Maximum likelihood estimates based on item calibrations in table 1.

** The severity of food insecurity in households that affirmed no items (raw score = 0) cannot be estimated based only on item calibrations. These households are more food secure than those that affirmed one item, but the size of the interval is unknown. Technically, the severity of food insecurity in households that affirmed all 8 items is also unknown, but given that this comprises only a small proportion of households, it is conventional to estimate their severity at a raw score of 7.5, as was done in this case.

The following prevalence estimates for the Isfahan sample are based on thresholds similar to those used in the United States and Canada and are for illustrative purposes only. A threshold for food insecurity of either 3+ (similar to that used by the United States Department of Agriculture) or 2+ (similar to that used by Health Canada) may be appropriate, depending on the consensus of national experts on nutrition and public policy. Depending on the threshold selected, either 45.8 percent of households (those with raw scores of 3 or higher) or 55.2 percent of households (those with raw scores of 2 or higher) may be classified as food insecure (i.e., included one or more food-insecure adults). If severe food insecurity is conceptualized as a condition in which adults either reported that they were hungry but did not eat, or lost weight, or did not eat for a whole day (those with raw scores of 6 or higher), then 11.6 percent of households in the Isfahan sample had severe food insecurity among adults. A less severe threshold (raw score of 5 or greater) would also include households in which adults ate less than they thought they should, even if they did not report being hungry. Based on that less severe threshold, 17.4 percent of households in the Isfahan sample had severe food insecurity among adults.

### Child scale

**Figure 2:**
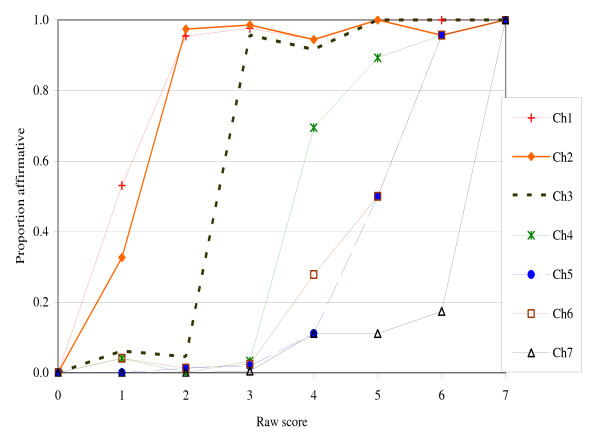
**Item responses by raw score, child items in Isfahan Food Security Pilot Survey**.

The child-referenced items were very consistently ordered. For example, item CH3, "children were not eating enough" was denied by almost all households with raw score 2 and affirmed by almost all households with raw score 3 (figure 2). The very low in fit of 0.52 for this item confirms its very high discrimination (table 4). For classification at the level of severity of this item, the response to this single item would perform almost as well as the 7-item scale. The infit statistic for item CH7 was slightly high (1.26). It may be possible to improve this item, but the high infit reflects just two or three out-of-order responses, since the numbers of cases with raw scores 4 and higher were small in this study. The average item discrimination of the Isfahan child scale is similar to, or somewhat higher than, that of the U.S. scale.

**Table 4:** Response characteristics, item calibrations, and item-fit statistics of items in the Isfahan Children's Food Security Scale

Item *	Affirmative responses(%)**	Item calibration***	SE****	Item infit	Item outfit
CH1 Few kinds of low-cost food	49.0	3.24	0.134	1.06	16.46
CH2 Could not afford nutritious meals	48.4	3.40	0.128	0.83	109.28
CH3 Not eating enough	32.0	5.86	0.094	0.52	0.98
CH4 Reduced size of meals	10.3	8.64	0.118	0.70	12.46
CH5 Skipped meals	6.8	9.53	0.145	0.85	4.97
CH6 Hungry	7.8	9.26	0.137	1.03	34.62
CH7 Did not eat whole day	3.3	11.03	0.222	1.26	1.16
					
Mean (item calibrations)		7.28			
Standard deviation (item calibrations)		2.88			
Discrimination parameter		1.50			
Number of cases *****	990	501	501	501	501

*The full wording of each question specified the time reference (previous 12 months) and specified that the condition occurred because of a lack of money for food.

**Percent affirmative responses(%) of all households with children

***Item calibration indicates the severity of the item. The calibrations for the child scale were estimated with the mean and standard deviation constrained equal to the mean and standard deviation of the same items when scaled jointly with the adult items. This provides the most meaningful comparison of the severity of child items relative to adult items. The discrimination parameter of 1.5 for the child scale required to effect this transformation indicates that the discrimination of the child items is considerably higher when scaled alone than when scaled jointly with the adult items – primarily a result of the differing relationships between adult and child responses across households with children of different ages.

****Standard error of estimation of item calibration

***** The scaling sample omits households that affirmed no items or that affirmed all items, since those responses do not provide information about the severity of the items relative to one another.

To facilitate comparisons of the severities of adult- and child-referenced items, the calibrations of the child items as presented in table 4 have been adjusted by a linear transformation to equate the mean and standard deviation to those for the same items when they were scaled jointly with the adult items. (The scaling analysis of the combined set of items is not shown.) Items describing food insecure conditions of children are more severe (less likely to be reported) than those describing similar conditions among adults. For example, item CH6 (children were hungry) is 1.3 logistic units more severe than the similar adult item (AD6); and item CH7 (child did not eat for whole day) is 1.5 logistic units more severe than the similar adult item (AD8).

**Figure 3:**
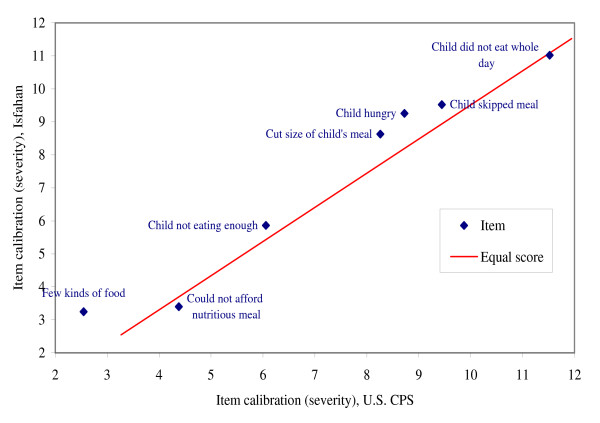
**Comparison of calibrations (severity parameter estimates) of child items in the Isfahan Food Security Survey versus the U.S. Current Population Survey Food Security supplement**. Note: For this comparison, the calibrations of the items estimated from the U.S. CPS-FSS data were adjusted by a linear transformation to equate the mean and standard deviation of the item calibrations to those estimated from the Isfahan data.

Figure 3 plots the item calibrations for the Isfahan child items against those from the U.S. CPS-FSS. The items are in the same order of severity in the two surveys. The calibrations of the first two items differ only slightly in the Isfahan data, while they differ substantially in the U.S. data. With that exception, the relative severities of the items are similar in the two surveys. Thus, for children as well as adults, the character of the phenomenon of food insecurity (although not necessarily its prevalence) appears to be quite similar in the Isfahan and U.S. populations.

The assessment of conditional independence, or dimensionality (not shown), indicated that the 7 child items represent, essentially, a single dimension.

Using thresholds consistent with those applied in the United States and Canada, 47.8 percent of households in the sample (those with raw scores 2 and higher) had food insecurity among children, including 7.3 percent with severe food insecurity among children (those with raw scores 5 and higher; table 5). (The more severe range of food insecurity among children is described as "severe food insecurity" by Health Canada and as "very low food security among children" by the United States Department of Agriculture. Prior to 2006, the category was described by the U.S. Department of Agriculture as "food insecure with hunger among children.")

**Table 5:** Measured values of severity of food insecurity among children by raw score on Isfahan children's food security scale, and percentage of households with each raw score

Raw score	Household score*	Measurement error	Percent of households***	Cumulative percent of households***
0	Unknown**	NA	47.27	47.27
1	3.29	0.93	4.95	52.22
2	4.85	1.18	15.56	67.78
3	7.07	1.29	21.31	89.09
4	8.64	0.82	3.64	92.73
5	9.55	0.77	2.83	95.56
6	10.59	0.92	2.32	97.88
7	11.36**	1.13	2.12	100.00

* Maximum likelihood estimates based on item calibrations in table 1.

** The severity of children's food insecurity in households that affirmed no items (raw score = 0) cannot be estimated based only on item calibrations. The children in these households are more food secure than those in households that affirmed one item, but the size of the interval is unknown. Technically, the severity of children's food insecurity in households that affirmed all 7 items is also unknown, but given that this comprises only a small proportion of households with children, it is conventional to estimate their severity at a raw score of 6.5, as was done in this case.

*** Households with no children present were omitted from these calculations.

The measured range of the Isfahan 7-item child scale is from 3.29 to 10.59, a total range of about 7.3 units. Considering the standard errors of measurement, this range is adequate differentiate three ranges of severity.

To assess the food security of households with children, conditions among both adults and children may be taken into account. We cross-tabulated the 990 households with children in the Isfahan Food Security Survey on the adult and child scales to examine these relationships. For this analysis, we used the more severe thresholds on the adult scale (3+ for food insecure and 6+ for severe food insecurity). Based on these classifications both adults and children were food secure in 41.8 percent of households with children. In 58.2 percent, either adults or children or both were food-insecure, including 10.4 percent in which only adults were food insecure, 11.5 percent in which only children were food insecure, and 36.3 percent in which both adults and children were food insecure. In 14.2 percent of households, either adults or children or both were severely food insecure, including 7.0 percent in which only adults were severely food insecure, 2.3 percent in which only children were severely food insecure, and 4.9 percent in which both adults and children were severely food insecure.

## Discussion

The U.S. Household Food Security Survey Module as translated and implemented in the Isfahan Food Security Survey provides internally valid household-level measures of food insecurity among adults and children. The dispersion of items across a considerable range of severity along with acceptable item-fit statistics indicates high average item discrimination and good fit to the Rasch measurement model. It is expected that a household that says "yes" to an item will say "yes" to most of the items that are less severe. Similarly, a household that says "no" to an item is expected to say "no" to most items that are more severe. These patterns are not expected to be absolute, but only probabilistically true. The dispersion (standard deviation) of item severity parameters is a measure of the extent to which these expected response patterns predominate. Low average discrimination might indicate that the questions were not consistently understood, or that respondents did not take the survey seriously, or that interviewers were careless in reading questions or recording responses. The consistency of response patterns in the Isfahan data is evidence that none of these problems was present at any serious level in this survey.

Two substantive changes were made from the U.S. methodology: three follow up questions in the U.S. module that ask how often specific reported conditions occurred during the previous 12 months added very little information and were omitted from the measures, and separate scales for measuring the food security of adults and children were used rather than a single scale combining adult and child items. Items describing food insecure conditions of children are more severe than those describing similar conditions among adults. These differences represent the extent to which children are generally protected from adverse consequences of the household's food insecurity even at the cost of greater hardship for the adults. However, these relationships are not consistent across households. They depend on household characteristics – primarily on the ages of children. This dependency is evidenced by the much lower discrimination of the child items when they are scaled with the adult items. Because of this dependency, we recommend use of separate scales to measure the food security of adults and children in Iran.

Food insecurity, as measured by the adapted module, appears to be essentially the same phenomenon as that measured by the corresponding module in the United States. That is, with minor exceptions, food insecurity is experienced, managed, and described in terms of the same experiences and behaviors, and in the same order of severity, in the two countries. The higher severity of item AD1 in the Isfahan study indicates that, controlling for responses to all other questions, this condition is less likely to be reported by Isfahani households than by U.S. households – that is, that it represents a more severe condition. The opposite is true for item AD3 (could not afford to eat nutritious meals). These differences may result from inexact translation. The questions may refer to somewhat different objective conditions in the two languages. Alternatively, food insecurity may be experienced and managed somewhat differently in the Iran and the United States, with the result that the conditions to which these questions refer relate to food insecurity somewhat differently in the two countries. The validity of the Isfahan scale does not depend on the measure being the same as that used in the U.S. However, if comparisons are made, it should be kept in mind that raw scores in the less severe range on the Isfahan scale will represent only approximately equivalent conditions to those represented the same raw scores on the U.S. scale.

Studies to validate the HFSSM have been conducted in several other countries: Bolivia [14], Brazil [15], Colombia [30], Mexico City [31], Trinidad and Tobago [32], Ecuador[33], and Canada [16]. Different methods and internal validity tests were used in these studies; the results from our research in Isfahan are similar to them in confirming the appropriateness of the HFSSM to determine the severity of food insecurity of households.

In Bolivia [14] and Brazil [15], criterion validity of the tool was established using food expenditure and intake respectively. The studies included both rural and urban areas, but had relatively small sample sizes (n = 125 and 327).

Rasch-model-based analyses were used to assess food security measures in Colombia [30] and Ecuador [33]. Acceptable internal validity of the measures were found both in the large regionally representative sample (n = 1624) in Colombia and the smaller rural sample in Ecuador The Rasch model fit the Isfahan data somewhat better than that from the study in Ecuador [33] using 54 households of rural community. Ecuadorian researchers considered in fit values within a range of 0.6 to 1.4 to be acceptable, whereas in Isfahan, no item had an in fit higher than 1.3, and only one (CH3) had an in fit below 0.7. This difference may be related to the culture in different areas and small and a small heterogeneous sample in the Ecuadorian study.

As with the Isfahan study, adult and child items were analyzed separately in the Trinidad and Tobago study [32]. Researchers assessed both 1- and 2-parameter models and concluded that for both adult and child measures a single-parameter logistic model fit the data adequately.

Although overall performance of the module was adequate, high outfit statistics for several items and low infit statistics for two items suggest that improvement of some items should be attempted prior to widespread policy-oriented use of the module. Specifically, adult items AD1, AD3, AD4, and AD7 and child items CH1, CH2, CH4, and CH6 may benefit from further examination using qualitative methods. The high outfit statistics for these items indicates a larger-than-expected proportion of highly improbable responses. Highly improbable responses include either an affirmative response to a very severe item by a respondent who denied most or all of the less severe items, or a negative response to a low-severity item by a respondent who affirmed several more severe items. It should be noted, however, that outfit statistics are sensitive to even a very small proportion of such responses, which may result from momentary lapses of attention by respondents or miscoding by interviewers. Even the very high outfit of item CH2 (109.28) resulted almost entirely from responses of three households out of the scaling sample of 501. In a different survey context or a somewhat different or larger sample, some of these problems may disappear. Nevertheless, further cognitive testing of these questions should be undertaken to improve consistency of comprehension if possible.

The low infit statistics (below 0.8) for AD1 and AD6 indicate that these conditions are more consistently and strongly related to food insecurity than the other items. Improving the other items so as to reduce the proportion of erratic responses will improve the overall model fit, and thereby increase the infit statistics of these items.

In further developing the module, attention may be given to whether AD1 and AD2 questions are really asking about the same condition. If so, could one be changed to capture a slightly different condition (for example, anxiety in AD1 and actual depletion of food stores in AD2)? If not, it may be preferable to drop one of them. Questionnaire layout issues (such as page break or word use) should also be examined to be sure these two items are not inadvertently grouped in the way they are administered.

## Abbreviations

AD: Adult; CH: Child; US-HFSSM: U.S Household Food Security Survey Module.

## Competing interests

Funding of this work is supported by Vice Chancellor for Research in Isfahan University of Medical Sciences. We have no financial or other relationships that might lead to a conflict of interest.

## Authors' contributions

MR as Main Researcher participated in all parts from designing proposal and data collection to writing article and submission. MN contributed in data analysis, conclusion of the results and article writing. AS had a contribution in manuscript edition and submission. MHE was consulted for designing the proposal. Finally, the manuscript has been read and approved by all the authors.

## Disclaimer

Views expressed are those of the authors and may not be attributed to the Economic Research.

Service or the United States Department of Agriculture.
